# Impact of early genomic prediction for recurrent selection in an upland rice synthetic population

**DOI:** 10.1093/g3journal/jkab320

**Published:** 2021-09-08

**Authors:** Cédric Baertschi, Tuong-Vi Cao, Jérôme Bartholomé, Yolima Ospina, Constanza Quintero, Julien Frouin, Jean-Marc Bouvet, Cécile Grenier

**Affiliations:** 1 CIRAD, UMR AGAP Institut, F-34398 Montpellier, France; 2 UMR AGAP Institut, Univ Montpellier, CIRAD, INRAE, Institut Agro, F-34398 Montpellier, France; 3 Rice Breeding Platform, International Rice Research Institute, Metro Manila, Philippines; 4 Alliance Bioversity-CIAT, Recta Palmira Cali, Colombia; 5 CIRAD, Dispositif de Recherche et d’Enseignement en Partenariat “Forêts et Biodiversité à Madagascar”, Antananarivo, Madagascar

**Keywords:** rice, recurrent selection, genomic prediction, GxE, grain zinc concentration

## Abstract

Population breeding through recurrent selection is based on the repetition of evaluation and recombination among best-selected individuals. In this type of breeding strategy, early evaluation of selection candidates combined with genomic prediction could substantially shorten the breeding cycle length, thus increasing the rate of genetic gain. The objective of this study was to optimize early genomic prediction in an upland rice (*Oryza sativa* L.) synthetic population improved through recurrent selection via shuttle breeding in two sites. To this end, we used genomic prediction on 334 S_0_ genotypes evaluated with early generation progeny testing (S_0:2_ and S_0:3_) across two sites. Four traits were measured (plant height, days to flowering, grain yield, and grain zinc concentration) and the predictive ability was assessed for the target site. For days to flowering and plant height, which correlate well among sites (0.51–0.62), an increase of up to 0.4 in predictive ability was observed when the model was trained using the two sites. For grain zinc concentration, adding the phenotype of the predicted lines in the nontarget site to the model improved the predictive ability (0.51 with two-site and 0.31 with single-site model), whereas for grain yield the gain was less (0.42 with two-site and 0.35 with single-site calibration). Through these results, we found a good opportunity to optimize the genomic recurrent selection scheme and maximize the use of resources by performing early progeny testing in two sites for traits with best expression and/or relevance in each specific environment.

## Introduction

Population improvement strategies are recognized as methods to exploit the genetic diversity of a crop and enrich the genetic basis of breeding programs. In rice, population breeding through recurrent selection (RS) was suggested as a valuable option in countering the decline in genetic diversity among the improved rice germplasm from Latin America and the Caribbean (LAC; Cuevas-Pérez *et al.* 1992; Guimarães *et al.* 1996). RS in rice started in South America in 1985 (Taillebois and Guimarães 1989) and later spread to most of the continent through a Food and Agriculture Organization funded initiative (Châtel *et al.* 2005; Martínez *et al.* 2014). In the region, RS was applied to rice synthetic populations, each composed of several elite materials, carefully chosen as founders, which had intercrossed for various generations (Guimarães 2005). Following recurrent cycles of selection and recombination, several thousand S_0_ plants (S being used here to define the number of selfing cycles) are available for use in the breeding program either as new parents for population improvement or as S_0_ progenies for variety development. The particularity of RS breeding in rice as performed in various countries in LAC is that it uses a recessive nuclear male sterility (*ms*) gene to facilitate outcrossing (reviewed in Frouin *et al.* 2014). This gene allows random recombination among a large number of parental plants at each cycle. Different ways are used to improve populations carrying the *ms* gene (Châtel and Guimarães 1997). The most common practice is to evaluate a moderate number (200–300) of candidates randomly drawn from the synthetic population. The evaluation of the candidates is then performed through progeny testing, with more or less fixed families (S_0:2_, S_0:3_, or S_0:4_ depending on the trait and required experimental design, obtained through several cycles of inbreeding and bulk harvest). Subsequently, parental lines are selected to be used for the next recombination cycle. Among others, two compromises have to be made that have a direct impact on the genetic gain achieved by the RS breeding scheme: (1) the number of candidate units evaluated through progeny testing with direct impact on the selection intensity and; (2) the required degree of fixation of those progenies prior to phenotyping, which would affect the breeding cycle length and influence the precision of genetic variance estimates.

Since its introduction by Meuwissen *et al.* (2001), genomic prediction (GP) has been widely adopted by animal and plant breeders alike. By allowing rapid selection of superior genotypes and accelerating the breeding cycle, GP has shown great potential since the advent of this new breeding paradigm in crop species in 2007 (Bernardo and Yu 2007). The value of GP in the context of RS is fairly evident as the selection based on genomic estimated breeding value (GEBV) can be applied to a very large population of genotyped entries through the calibration of a prediction model performed on a reduced set of training units. Furthermore, the average progeny phenotypic values associated with the genomic matrix of the respective S_0_ individuals could allow a more precise estimate of the genetic variation in the case of early generation segregating candidate units. GP was simulated on multiparent populations (Heffner *et al.* 2011; Guo *et al.* 2012; Bian and Holland 2017; Allier *et al.* 2020a) directly related or not to a breeding program to assess the potential use of GP in genetic improvement through RS. However, few simulation studies have assessed the potential value of GP for crop synthetic populations (Müller *et al.* 2017, 2018; Schopp *et al.* 2017). Theoretically and through simulation approaches, recurrent genomic selection has the particular advantage of managing both the genetic gain and the maintenance of genetic diversity in the breeding program (Gorjanc *et al.* 2018; Allier *et al.* 2020b). In a simulated wheat breeding program, the inclusion of a step of population improvement with rapid recycling of early material proved to be greatly superior in terms of genetic gain compared with a program relying solely on biparental crosses between elite material to generate diversity (Gaynor *et al.* 2017). Similarly, recurrent genomic selection in soybean (Ramasubramanian and Beavis, 2020) and maize (Zhang *et al.* 2017) showed the long-term potential of RS combined with GP. GP has already been applied to material from RS for rice in single (Grenier *et al.* 2015; Morais Júnior *et al.* 2017) and multi-environment contexts (Morais Júnior *et al.* 2018). In these studies, the results showed relatively good predictive ability (PA) for various simple and complex traits such as plant height (PH), flowering date (FL), and grain yield (YLD). In both cases, however, the calibrations were based on material that underwent some degrees of fixation through plant selection and a few cycles of selfing. A significant jump in efficiency in these schemes is expected by calibrating on early generation candidates from S_0_ progenies to save time in building the models and to accelerate the recycling of the selected germplasm.

GP integrating genotype by environment interaction (GxE) has proven successful, showing greater PA than the single environment prediction, provided environments are positively correlated. An approach to multi-environment GP was proposed and applied by Burgueño *et al.* (2012) where the authors modeled the environment and genotype covariance structure and used it within a mixed model framework. Later, GxE was incorporated in a GP model by separately capturing the main marker effect, common to all environments, and an environment-specific marker effect (Lopez-Cruz *et al.* 2015). This method is easy to implement and showed good results for wheat breeding under multiple environmental conditions. Additionally, it has the advantage that it enables working with different genotype covariance structures. Genotype covariances based on either a linear kernel (GxE GBLUP) or a Gaussian kernel (GxE RKHS) have been tested, and the Gaussian kernel allows a more flexible structure than the linear kernel and potentially better prediction (Cuevas *et al.* 2016). To optimize calibration with the multi-environment data, various strategies of genome-based models including GxE were proposed (Jarquín *et al.* 2020). The authors compared different partitioning of the calibration sets among the multiple sites where the population was tested, with different degree of overlapping of the genotypes between environments. Sparse testing designs in which subset of the genotypes are tested in each location was presented as a method to reduce the experimental effort and optimize the use of breeding program resources.

This study was conducted in the context of a collaborative rice breeding program between CIAT (International Center for Tropical Agriculture, member of the CGIAR centers) and Cirad (French Agricultural Research Centre for International Development). The CIAT-Cirad rice breeding program has historically conducted population development and improvement through RS. Its current RS program based on progeny testing is conducted in two locations; at CIAT-HQ in Palmira, where rice is cultivated all year round under irrigated conditions, and in Santa Rosa (SRO), an experimental site where rice is grown under rainfed conditions during the main cropping season. Although aiming to implement early GP in our RS scheme, we were also interested in making optimal use of all the data gathered in both locations (target and not target) for the breeding program. The main objective was to evaluate the PA of the GP model including the GxE interaction to obtain reliable estimates of the breeding value of selection candidates in the target site.

## Materials and methods

### Development of PCT27 population

**Figure 1:**
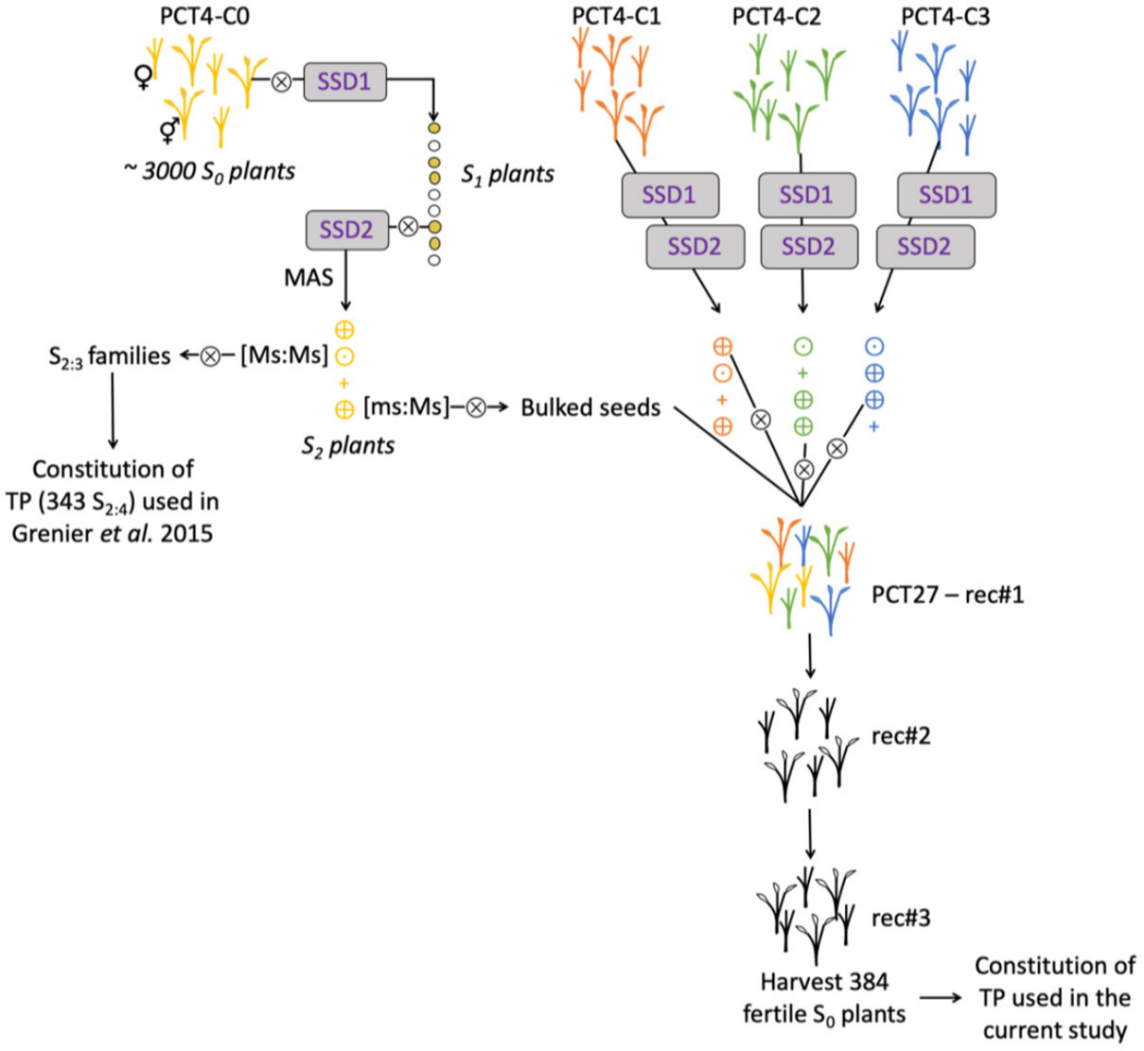
Process followed for the development of the PCT27 population. Populations PCT4-C0, PCT4-C1, PCT4-C2 and PCT4-C3 were described in Grenier et al. (2015). Each population contains about 3,000 plants with half male fertile plants (⚥) that can be selfed and half male sterile plants (♀). “SSD” is the single descend method of generation advance applied to 100 male fertile plants per population.⊗ indicates the selfing process. The “MAS” (marker-assisted selection) process was performed for the selection of S_2_ plants based on genotypic profile at the *ms* gene. Genotyped plants are symbolized as + for plants with the [ms:ms] genotype, ⨁ for the [ms:Ms] genotype and ⊙ for the [Ms:Ms] genotype. “rec” are recombination cycles performed by harvesting all male sterile plants from the population without any selection pressure. For PCT27—rec#1 this first recombination cycle was done among the progenies of 35 families randomly extracted among the four populations.

The genetic material used in this study belongs to the tropical japonica group of cultivated rice (*Oryza sativa* L.). Several synthetic populations developed in the CIAT-Cirad rice breeding program were improved for adaptation to upland ecosystems and acid soils. In 2015, Grenier *et al.* (2015) used a training set defined with 348 S_2:4_ lines derived from four populations to study the potential of GP in an RS scheme. Of the 348 families at the S_2_ generation, marker-assisted-selection for the *ms* gene (Frouin *et al.* 2014) helped to select [ms:MS] male fertile plants in 35 randomly sampled families. One single plant per family was selfed, and the seeds of each of 35 plants were mixed in equal proportion to generate a candidate population hereafter referred to as PCT27 (Figure 1). Two recombination cycles were performed at CIAT-HQ in Palmira under irrigated conditions in a bundled field isolated from other rice experimental plots by at least 50 m to avoid pollen contamination and without any selection pressure. At each cycle, a population of about 3000 plants was established with male sterile and male fertile plants randomly distributed within the plot. The recombining units were then collected by harvesting male sterile plants pollinated by any male fertile plants in the vicinity. At the third cycle of recombination, 334 S_0_ fertile plants were randomly extracted from the population to constitute our reference population. All entries were advanced to the S_0:2_ and then S_0:3_ generation by bulk harvesting seeds from 15 to 20 male fertile plants per line per generation. Additionally, 50 temporal checks from the same population were also advanced by bulk method to the generations S_0:2_ and S_0:3_ and were used to test the generation effect and the year effect within the site. The terms line and genotype were used indifferently in this work to refer to the S_0_ plants and their bulked offspring at either generation S_0:2_ or S_0:3_ if specified.

### Genotyping

Leaf tissues were sampled on the 334 S_0_ plants and DNA extraction was performed as in Grenier *et al.* 2015. Genotyping was done by genotyping-by-sequencing (GBS) approach (Elshire *et al.* 2011). The detailed method is described in Appendix A and the genetic characterization of the population can be seen in Supplementary Tables and Figures. As a result of the genotyping and subsequent genetic analysis, the population was characterized by 9928 SNP markers fairly well distributed among the 12 rice chromosomes (Supplementary Table S1 and Figure S1). The MAF distribution among the 334 S_0_ reflects a population where rare alleles were not depleted, which fits well with long-term objectives of a population breeding program (Supplementary Figure S2). The degree of allelic fixation varied greatly between the genotypes but remained relatively low for individuals at the S_0_ generation (Supplementary Table S1). Considering the rather large average LD (Supplementary Table S2) and the slow LD decay observed (Supplementary Figure S3), the average marker density (1 SNP every 40 kb) was considered good enough to allow the capture of all linked QTLs with the SNP matrix in hand. The whole population was characterized, with a total absence of structure, which provides a good base for setting up a GP scheme through CV (Supplementary Figure S4).

### Field trial and phenotyping

Field phenotyping was performed at two locations in Colombia. One site was an experimental field at CIAT-HQ in Palmira (PAL) located in the Valle del Cauca, Colombia (3.50° N–76.35° W, 1000 masl). At this location, rice evaluation trials are conducted under irrigated conditions and can be performed all year round due to favorable environmental conditions and good water availability that enable the irrigation scheme throughout the crop cycle. As it is not a rice prone area, no severe disease pressure is naturally present, and the rice crop usually expresses its full potential. On the other hand, SRO is an experimental site, owned by the Colombian National Federation of rice growers (Fedearroz) located in the Oriental plains of Colombia, in the department of Meta, Colombia (4.03° N–73.48° W, 300 masl). At this site, the rice crop is established through direct seeding and the trials are conducted under rainfed conditions during the main cropping season, between May and September. The predominance of rice cultivation in this area, the climatic conditions of hot and humid summers during the main growing season and the natural occurrence of various strains of pathogens (bacterial, fungal, or viral) make this site a hot spot for disease screening.

Four trials were conducted during two consecutive years, 2017 and 2018, using different semesters for each location. Field trials were established in PAL on December 4, 2017 and December 10, 2018 and in SRO on December 5, 2017 and May 30, 2018. At each site, the experimental design followed a lattice with 16 blocks and three repetitions. The 50 temporal check lines (only S_0:2_ in the 2017 trials and S_0:2_ and S_0:3_ lines in the 2018 trials) were randomly distributed across the design within each repetition of the two sites and 2-year trials. In PAL, the trials were established after transplanting 3-week-old seedlings in a bundled field. The plot size was two rows of 17 plants with 25 cm between plants and between rows. Fertilizer application followed a split application with N, P and K nutrients added at 25 and 35 days after transplanting. Irrigation was maintained continuously in order to ensure a 25 cm layer of water in the field until a week prior to the crop maturation period. In SRO, the trials were established by direct sowing of two 4-m long rows, spaced by 26 cm at a density of one gram of seed per linear meter. Split fertilizer application was performed according to the recommended application for growing tropical japonica rice in upland soil conditions. Phytosanitary treatment was applied in SRO to prevent blast outbreaks. For all four trials, a similar design was applied, but with a different randomization.

Four traits were measured following the IRRI Standard Evaluation System (IRRI 2013) on the whole training population including the 50 temporal checks. FL was expressed as the number of days after crop establishment—being either the date after either transplantation (PAL) or sowing (SRO)— when 50% of the plants within a plot reached anthesis. PH was calculated as the average height measured in centimeters of five plants with their panicle extended. YLD was obtained by weighing the grains collected within each plot after discarding the plants at the start and end of each plot. For each harvested plot, percent humidity was measured and used to correct the weight of collected grains, expressed in grams per plot, for a relative humidity of 14%. The YLD value was neither adjusted for the plot size nor for the count of fertile plants. The grain zinc concentration (ZN), expressed in parts per million, was measured on a subsample of collected grains polished in Teflon equipment, using energy dispersive X-ray fluorescence spectrometry (X-supreme 8000, Oxford Instrument, Shanghai, CN) available at the CIAT-HQ Nutritional Laboratory. The exact same procedure was used for generation S_0:2_ in 2017 and generation S_0:3_ in 2018.

The 50 temporal checks were phenotyped as S_0:2_ in 2017 and as S_0:2_ and S_0:3_ in 2018. This allowed measurement of the non-confounded year within site effect on the S_0:2_ and the generation effect in 2018 by analyzing the data from the S_0:2_ and S_0:3_ lines as presented in Appendix B.

### Statistical models for GP

Raw data were visually explored for outliers as described in Appendix C. Based on clean data, Pearson’s correlation between phenotypic BLUPs obtained in PAL and SRO was computed for generations S_0:2_ and S_0:3_ using the 334 S_0_ families phenotyped in both generations.

All the models were estimated using ASReml-R v3.0 (Butler *et al.* 2007). GP was done independently in each generation. For single-site calibration, the following model was used:

(Model 1) $$Y_{\mathrm{ijk}}=\mu +r_{i}+b{\left(r\right)}_{ij}+g_{k}+\varepsilon_{\mathrm{ijk}}$$

The fixed effects were the intercept μ and the replicate effect $r_{i}$. The random part was composed of the block effect $b_{ij}$ nested in replicate with distribution $b∼N\left(0,I\sigma_{b}^{2}\right)$, the genotype effect $g_{k}$ that represents the progeny means with distribution $g∼N\left(0,M\sigma_{g}^{2}\right)$ and the residual $\varepsilon_{\mathrm{ijk}}$ with $\varepsilon ∼N(0,I\sigma_{\varepsilon }^{2}$).

The variance $\sigma_{b}^{2}$ is associated with the blocks, while $\sigma_{g}^{2}$ and $\sigma_{\varepsilon }^{2}$ are the genotypic and error variances, respectively. The two variance–covariance matrices used are I for the identity matrix and M representing the genotype variance–covariance computed according to either of the two prediction methods described below.

For the two-site approach the following model was used:

(Model 2) $$Y_{\mathrm{ijkl}}=\mu +s_{i}+r{\left(s\right)}_{ij}+b{\left(r\left(s\right)\right)}_{\mathrm{ijk}}+g_{l}+{gs}_{il}+\varepsilon_{\mathrm{ijkl}}$$

The fixed effects were the same as for Model 1, with an additional fixed site effect $s_{i}$. The random part of Model 1 was completed with the genotype (progeny means) by site interaction ${gs}_{il}$ with distribution $gs∼N\left(0,\left[\begin{array}{c} \begin{array}{cc} M_{\mathrm{PAL}}^{}\;\sigma_{\mathrm{gsPAL}}^{2} & \;0 \end{array}\\ \begin{array}{cc} 0 & {\;M}_{\mathrm{SRO}}^{} \end{array}\;\sigma_{\mathrm{gsSRO}}^{2} \end{array}\right]\right)\;$and the residual $\varepsilon_{\mathrm{ijkl}}$ with distribution $\varepsilon ∼N\left(0,\;I⨂\left[\begin{array}{c} \begin{array}{cc} \sigma_{\varepsilon \mathrm{PAL}}^{2} & \;0 \end{array}\\ \begin{array}{cc} 0 & \;\sigma_{\varepsilon \mathrm{SRO}}^{2} \end{array} \end{array}\right]]\right)$.

In addition to the three variances described in Model 1, Model 2 includes two site-specific genotype by site interaction variances $\sigma_{\mathrm{gsPAL}}^{2}$ and $\sigma_{\mathrm{gsSRO}}^{2}$ as well as two site-specific error variances $\sigma_{\varepsilon \mathrm{PAL}}^{2}$ and $\sigma_{\varepsilon \mathrm{SRO}}^{2}$. The error variance–covariance is modeled by the Kronecker product of the identity matrix and the variances matrix.

To compute the variance structure (*M*) for the genotype effect and genotype by site interaction ($M_{\mathrm{PAL}},M_{\mathrm{SRO}}$), two different kernels were used. In the first approach, GBLUP, $M=M_{\mathrm{PAL}}=M_{\mathrm{SRO}}$, where M was based on the linear kernel $M=\frac{XX`}{N}$ (Lopez-Cruz *et al.* 2015), a proportional of the matrix proposed by VanRaden (2008) was used, with *X* being the genomic data with genotypes coded as *−1, 0, 1* and *N* the number of markers. The second approach, RKHS, was based on the reproducing kernel Hilbert space approach by Gianola and van Kaam (2008). Three different variance–covariance structures were computed: one for the complete data ${(M}_{0})$ and one for each site independently $\left(M_{\mathrm{PAL}\;},M_{\mathrm{SRO}}\right),$ all based on the Gaussian kernel $K_{e}\left(x_{me},x_{ne}\right)=\exp\left(-h_{i}∥x_{me}-x_{ne}{∥}^{2}\right)$, for $x_{me},x_{ne}$ being two marker genotype vectors and $\left(m,n\right)\in {\left\{1,\ldots ,N\right\}}^{2}$. The bandwidth *h* controls the decay rate of the correlation between the lines, smaller *h* giving a sharper correlogram. We computed *h* with the method proposed by Pérez-Elizalde *et al.* (2015) and the provided R function *marg.fun*. A gamma prior distribution for *h* was used, the shape parameter was set at 3 and the scale parameter set at 1.5. Three different bandwidth parameters were computed as the method relies partially on phenotypes, and hence yields different kernels depending on the site. New bandwidth parameters were estimated at each cross-validation (CV) cycle based on the BLUP-adjusted phenotypes of the sampled training set, as in Pérez-Elizalde *et al.* (2015). For both methods, the genotypic information was based on 9928.

Models 1 and 2 with identity matrix as variance–covariance matrices were used to compute broad sense heritability. *H*^2^ at trial level (generation within site) was used as a measure for repeatability and computed using the formula:

(1) $$H^{2}=\frac{\sigma_{g}^{2}}{\sigma_{g}^{2}\;+\;\frac{\sigma_{\varepsilon }^{2}}{NR}}$$

, where *NR* represents the harmonic mean number of plots per genotype. The global *H*^2^ per generation (considering the two sites for each generation) was computed with Model 2 following the formula:

(Eq. 2) $$H^{2}=\frac{\sigma_{g}^{2}}{\sigma_{g}^{2}+\frac{\sigma_{\mathrm{gsPAL}}^{2}+\sigma_{\mathrm{gsSRO}}^{2}}{NE}+\frac{\sigma_{\varepsilon \mathrm{PAL}}^{2}+\sigma_{\varepsilon \mathrm{SRO}}^{2}}{NR}}$$

, where *NE* is the harmonic mean of the number of sites per genotype, $\sigma_{\mathrm{gsPAL}}^{2}$ and $\sigma_{\mathrm{gsSRO}}^{2}$ are the genotype by site interaction variance for each site, *NR* is the number of plots per genotype across both sites and $\sigma_{\varepsilon \mathrm{PAL}}^{2}$ and $\sigma_{\varepsilon \mathrm{SRO}}^{2}$ are the residual variances for respectively PAL and SRO, respectively. Hence, *H*^2^ was based on the genetic variance and the mean interaction variance and mean residual variance across the two sites (Holland *et al.* 2003, Isik *et al.* 2017). $H^{2}$ was computed with the function *pin* from R-package *nadiv*, adapted to use external harmonic means.

### CV schemes for evaluating PA

**Figure 2:**
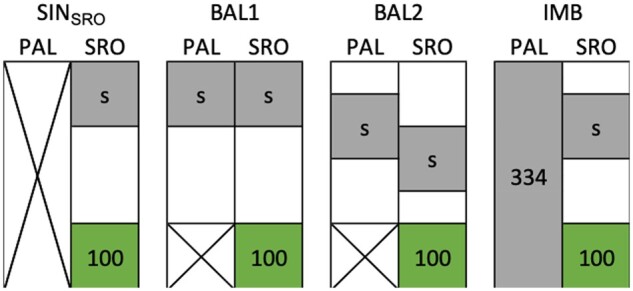
The four scenarios of CVs to evaluate the prediction accuracy in Santa Rosa (SRO). The first scenario (SIN_SRO_) uses phenotypic information from a single site, whereas the three others include Palmira (PAL) phenotypes in two-site models. In the latter case, the level of information between locations is either balanced (BAL) or imbalanced (IMB). The gray area represents the genotypes included in the training set with a varying size “s” to calibrate the model and the green area represents the validation set fixed to 100 genotypes.

Several CV schemes were used with different partitioning of the population among the two sites (Figure 2 and Supplementary Table S3).

**Figure 3:**
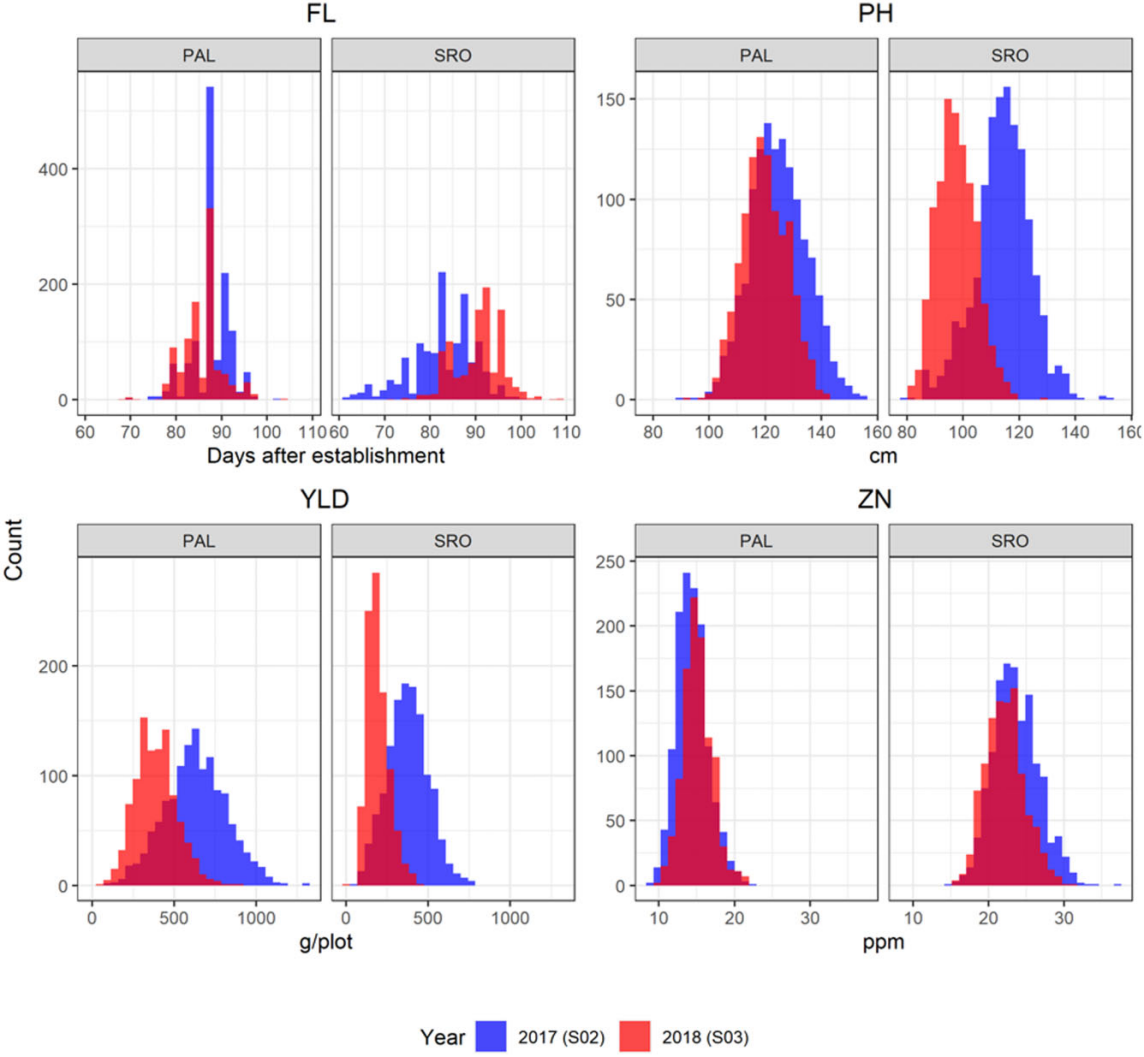
Histograms of the raw phenotypic values of the four traits: flowering day (FL), plant height (PH), grain yield per plot (YLD), and grain Zn concentration (ZN). The two environments: Palmira (PAL, irrigated) and Santa Rosa (SRO, rainfed) are represented. Outliers were discarded as presented in Appendix B.

In the first instance, only phenotypic data from the target site of selection SRO was considered (Figure 2). In that scenario, predictions were obtained based on Model 1 with a calibration based on a single site (SIN_SRO_). Various calibration set sizes (s) were tested, s ϵ {25, 50, 100, 200}.

For the two-site CV procedures, Model 2 was used. Calibrations were constructed with either a balanced (BAL) or imbalanced (IMB) representation of both sites. BAL1 represents a calibration method where both sites were represented by an equal number of phenotyped S_0_ families. Sets of “s” S_0_ were selected and their phenotypes in both sites were used for the training (Figure 2). This corresponds to a CV1 in Burgueño *et al.* (2012). For BAL2, “s” refers to the number of S_0_ families observed in SRO and in PAL, however, only a fraction of the families was observed in both sites (*i.e.*, the overlap), the remaining families being observed in only one of them. An overlap of 50% of the total number families included in the calibration was targeted. For “s” S_0_ families observed in both sites, the total number of S_0_ families was then $\frac{3}{2}\;$s. For the IMB scenario, the whole population was phenotyped in PAL and only a fraction of size “s” was phenotyped in SRO (Figure 2).

The same CV procedures were applied to each generation and with both GP models (GBLUP and RKHS). The GEBVs in SRO were obtained for the S_0_ included in the validation set, defined as the set for which no phenotype at SRO was recorded. In each scenario, 100 alternative samplings were performed for which the PA was measured as $\mathrm{cor}\left(\hat{Y},\mathrm{GEBV}\right)$. The reference $\hat{Y}$ was obtained with the complete SRO phenotypes using Model 1 and $M_{\;}=I$, I being an identity matrix and computed as ${\hat{Y}}_{k}=\mu +g_{k}$. GEBVs were obtained with the models including molecular information as ${\hat{Y}}_{k}=\mu +g_{k}$ for SIN_SRO_ or ${\hat{Y}}_{\mathrm{SRO},l}=\mu +s_{\mathrm{SRO}}+g_{l}$ for the other predictions (BAL1, BAL2, and IMB). For each scenario, the mean and the standard deviation of PA were computer on the 100 iterations.

To ensure that the variation in accuracy between the CV procedures was only due to the size differences in the training set, the correlations were always computed on the predictions for 100 genotypes randomly selected from the validation sets. However, for BAL2 with a training set size of 200, the validation set was reduced to 34 genotypes as those were the only genotypes with no phenotypic records that could be used for the validation with this strategy (Supplementary Table S3). The PA was still computed, but as the correlation was computed on only 34 points, the results must be considered with caution.

### Effects of the calibration parameters on PA

To investigate the response of the PA to the calibration parameters, linear models were fitted to the PA obtained from the 100 iterations with each scenario. Depending on the scenario, the independent variables were year, GP method, CV scenario, training set size, and all their combinations. Proportion of variance associated with one or more main effect, errors or interactions were estimated through the Eta^2^, as Eta^2^ = SSq_effect_/SSq_total_, where SSq_effect_ is the sum of squares for the effect under consideration and SSq_total_ is the total sum of squares of all effects, errors, and interactions in the ANOVA study. Throughout the text, this ratio is expressed as a percentage.

## Results

### Effect of sites and generations on the phenotypic performance

The phenotypic data were collected in two sites and on the same S_0_ progeny at two generations. In each site, the phenotyping was done in 2017 for 334 families at the S_0:2_ generation and in 2018 for the same 334 families at the S_0:3_ generation.

For most traits recorded in the two locations, the mean phenotypic values differed between sites (Table 1 and Figure 3). Although the differences between sites were moderate for FL and PH, they were large for YLD and ZN, with more than 60% change in the 2017 trials. The S_0:2_ families evaluated in 2017 had later flowering, shorter PH, lower yield and higher zinc concentration in SRO than in PAL. However, this tendency did not hold for the 2018 trials. The differences between sites in PH were greater at the 2018 trials, with taller S_0:3_ plants in SRO. For each trait, the spread of the data was consistent across site and year with 0.4–4 points of difference in the coefficient of variation. The highest coefficient of variation was observed for YLD in 2018 (34%), and was higher than in the 2017 trial (27%). The trait broad sense heritability (*H*^2^) at trial level showed large differences between traits and across sites and years. This measure of trial repeatability ranged from 0.52 for YLD in the PAL_2017 trial to 0.96 for FL in SRO_2017. Heritability was systematically higher in SRO than in PAL, and similar or slightly increased in the 2018 trials for all traits in all locations, but for FL and YLD measured in SRO.

**Table 1:** Descriptive values of the experiments in all trials (site × generation combinations) with mean, standard error (SE), coefficient of variation (*C*_var_), and broad sense heritability (*H*^2^) from Model 1

		S_0:2_ generation in 2017					
Trait ^*a*^	Site	mean	SE	min	max	*C*_var_	*H* ^2^ (SE)
FL	PAL	88.24	0.24	75	102	3.88	0.69 (0.03)
	SRO	82.17	0.37	61	96	7.93	0.96 (<0.01)
PH	PAL	125.62	0.62	88.4	155.4	7.76	0.61 (0.04)
	SRO	116.65	0.59	94.2	151.8	6.68	0.79 (0.02)
YLD	PAL	673.85	10.33	237.5	1311.5	24.07	0.52 (0.05)
	SRO	398.54	9.75	54.3	755.1	27.6	0.75 (0.02)
ZN	PAL	14.3	0.18	8.8	22	14.39	0.71 (0.03)
	SRO	23.8	0.21	15.9	37.1	12.64	0.81 (0.02)

		S_0:3_ generation in 2018					

FL	PAL	85.7	0.33	68	103	5.04	0.74 (0.02)
	SRO	90.54	0.36	72	108	5.76	0.78 (0.02)
PH	PAL	119.84	0.55	92.5	142.67	6.71	0.76 (0.02)
	SRO	97.63	0.53	80.8	128	7.09	0.80 (0.02)
YLD	PAL	387.54	8.3	54.6	901.1	32.23	0.56 (0.04)
	SRO	191.4	7.37	10.7	461.6	33.91	0.58 (0.04)
ZN	PAL	15.14	0.16	10.05	21.9	12.82	0.75 (0.02)
	SRO	22.21	0.18	15.3	30.8	11.51	0.81 (0.02)

a Traits are days to flowering (FL), plant height (PH), grain yield per plot (YLD), and grain Zn concentration (ZN)

As the year and the generation effect were confounded, 50 temporal checks were used to untangle the potential effects of generation and year. The significance of the fixed effect and variance decomposition among the 50 temporal checks showed that differences were exclusively due to year effect and neither a significant generation effect nor a significant genotype by generation interaction could be observed (Supplementary Table S4).

For each trait scored in each year, an analysis of the variance components was performed on the combined data from both sites using Model 2 (Table 2). The proportion of variance explained by the genotype effect was greater that of the combined genotype by site interaction effects from both sites (GxS_PAL_ and GxS_SRO_) only for FL in 2018, and PH recoded in both years. As a result, greater heritability was observed for these traits/year combination with *H*^2^ = 0.57, 0.50, and 0.62 for FL_2018, PH_2017, and PH_2018, respectively. The lowest genotype contribution to the explanation of variance was encountered for YLD, with large interaction effects and error effects associated with a particular site for each year, resulting in low *H*^2^ in both years (*H*^2^ = 0.19 and 0.11 in 2017 and 2018, respectively). For ZN, the genotype effect represented a third of the combined GxS interaction variances in both year trials, leading to similar and moderate *H*^2^ for both years (*H*^2^ = 0.38 and 0.40 for 2017 and 2018, respectively). The variance decomposition for each trait was coherent with the site correlation observed within years (Table 3). The highest correlations between SRO and PAL were observed for PH (*r* = 0.62) and FL (*r* = 0.62) in 2018. For the same traits in 2017, the correlations were lower (*r* = 0.55 and 0.51 for FL and PH, respectively). The site correlation was the lowest for YLD in both years (*r* between 0.13 and 0.20) and intermediate for ZN with comparable values in both years (*r* = 0.41 and 0.42 in 2017 and 2018, respectively).

**Table 2:** Variance decomposition and broad sense heritability (*H*^2^) from Model 2 by trait and generation

Trait ^*a*^	Variance component	S_0:2_ generation in 2017			S_0:3_ generation in 2018		
		Variance	Proportion	*H* ^2^ (SE)	Variance	Proportion	*H* ^2^ (SE)
FL	Genotype	4.92	0.11	0.25 (0.03)	7.86	0.22	0.57 (0.03)
	GxS_PAL_	<0.001	<0.001		<0.001	<0.001	
	GxS_SRO_	26.44	0.62		4.49	0.13	
	Bloc	0.93	0.02		1.89	0.05	
	Residual_PAL_	5.59	0.13		9.23	0.26	
	Residual_SRO_	4.93	0.12		12.4	0.35	
PH	Genotype	21.87	0.17	0.50 (0.04)	22.25	0.26	0.62 (0.03)
	GxS_PAL_	7.93	0.06		6.8	0.08	
	GxS_SRO_	7.95	0.06		3.14	0.04	
	Bloc	5.67	0.05		4.35	0.05	
	Residual_PAL_	57.48	0.46		29.9	0.34	
	Residual_SRO_	24.16	0.19		20.36	0.23	
YLD	Genotype	1,796.61	0.05	0.19 (0.05)	498.32	0.03	0.11 (0.05)
	GxS_PAL_	4,148.64	0.12		3,220.8	0.19	
	GxS_SRO_	3,919.93	0.12		540.88	0.03	
	Bloc	1,732.23	0.05		1,160.68	0.07	
	Residual_PAL_	16,676.45	0.49		9,301.29	0.53	
	Residual_SRO_	5,768.75	0.17		2,674.47	0.15	
ZN	Genotype	1.49	0.14	0.38 (0.04)	1.31	0.16	0.40 (0.04)
	GxS_PAL_	0.16	0.02		0.27	0.03	
	GxS_SRO_	3.05	0.29		2.28	0.27	
	Bloc	0.61	0.06		0.44	0.05	
	Residual_PAL_	2.02	0.19		1.62	0.19	
	Residual_SRO_	3.11	0.30		2.53	0.30	

GxS_PAL_ and GxS_SRO_ are the genotype by site interaction variances associated with PAL and SRO, respectively. Bloc stands for the variance associated with bloc within replicate within site. Residual_PAL_ and Residual_SRO_ are the residual variances associated with PAL and SRO, respectively.

a Traits are days to flowering (FL), plant height (PH), grain yield per plot (YLD), and grain Zn concentration (ZN)

**Table 3:** Pearson’s phenotypic correlations and *P*-value for each phenotypic trait (BLUPs obtained from Model 1) recorded in the two sites PAL and SRO within each year of field trial

Trait ^*a*^	S_0:2_ generation in 2017	S_0:3_ generation in 2018
FL	0.554 (<0.001)	0.624 (<0.001)
PH	0.509 (<0.001)	0.620 (<0.001)
YLD	0.206 (<0.001)	0.134 (0.014)
ZN	0.408 (<0.001)	0.424 (<0.001)

a Traits are days to flowering (FL), plant height (PH), grain yield per plot (YLD), and grain Zn concentration (ZN)

### Predictive abilities with calibration using single environment data

**Figure 4:**
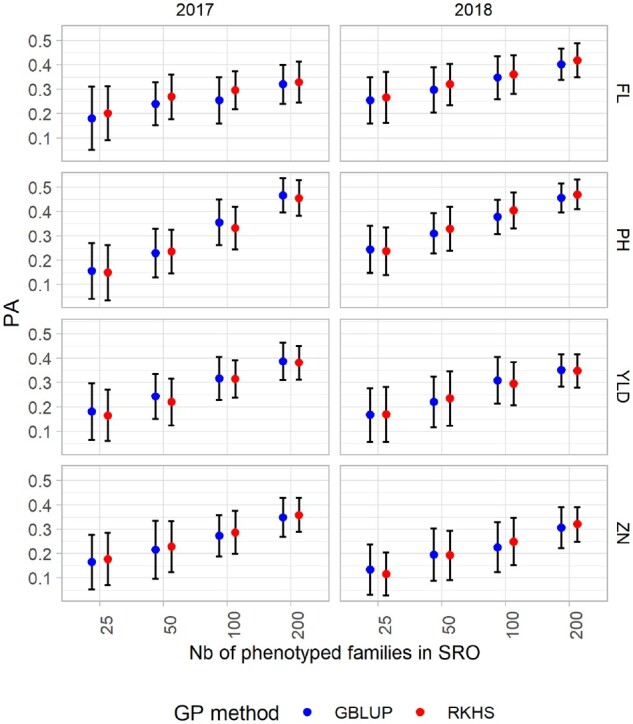
Mean predictive ability (PA) for the single-site model in Santa Rosa (SRO) for the four traits: flowering day (FL), plant height (PH), grain yield per plot (YLD), and grain Zn concentration (ZN), scored in 2 years (2017 and 2018). Four training set sizes (25, 50, 100, and 200) and two GP methods (GBLUP and RKHS) are considered. The bars represent the standard deviation.

The effects of different parameters used for the calibration of the model were first investigated for the PA from the single environment CV in SRO; SIN_SRO_ (Figure 4). Similar global average PA were achieved for all traits combining set sizes, years and GP methods (PA = 0.30, 0.33, 0.27, and 0.24 for FL, PH, YLD, and ZN, respectively; Supplementary Table S5). The linear model including all the factors taken individually, their first-order interaction and one second-order interaction explained 33–59% of the observed variation of PA (Table 4), indicating that a large proportion of the variability was due to the sampling of the CV method.

**Table 4:** Analysis by trait of the factors influencing the variability of the PA

		SIN_SRO_	
Trait ^*a*^	Factor ^*b*^	Eta^2^	*R* ^2^
FL	Year	0.105	0.333
	GP method	0.009	
	Set size	0.215	
	Year: GP method	0.000	
	Year: set size	0.003	
	GP method: set size	0.001	
	Year: GP method: set size	0.001	
PH	Year	0.043	0.592
	GP method	0.000	
	Set size	0.529	
	Year: GP method	0.002	
	Year: set size	0.017	
	GP method: set size	0.001	
	Year: GP method: set size	0.001	
YLD	Year	0.004	0.395
	GP method	0.001	
	Set size	0.386	
	Year: GP method	0.001	
	Year: set size	0.003	
	GP method: Set size	0.000	
	Year: GP method: Set size	0.001	
ZN	Year	0.027	0.358
	GP method	0.001	
	Set size	0.327	
	Year: GP method	0.000	
	Year: set size	0.001	
	GP method: set size	0.001	
	Year: GP method: set size	0.001	

The results are for the CV SIN_SRO_ scenario. Eta^2^ is the proportion of variance associated with each effect and R^2^ is the coefficient of determination obtained from a linear model applied to the data from the 100 iterations (*n* = 1600).

a Traits are days to flowering (FL), plant height (PH), grain yield per plot (YLD), and grain Zn concentration (ZN).

b Factors are Year (2017 and 2018), GP method (GBLUP and RKHS) and set size (25, 50, 100, and 200).

The training set size accounted for most of the PA variance explained by the model for all the traits. The largest training set size greatly improved the PA for all the traits (Eta^2^ = 22%, 53%, 39%, and 33% for FL, PH, YLD, and ZN, respectively). The year factor described a lower proportion of the total explained variance, with a maximum of 11% of the explained variance in PA for FL. For all traits GP model explained only a very limited proportion (<1%) of the variance. For most traits (PH, YLD, and ZN), the average PA was greater when predictions were performed with the GBLUP model. For this reason, the rest of the article will focus on the results achieved with the GBLUP model. However, the results for RKHS can be found in Supplementary Material (Supplementary Table S8).

### Predictive abilities with calibration using single and two-environment data

**Figure 5:**
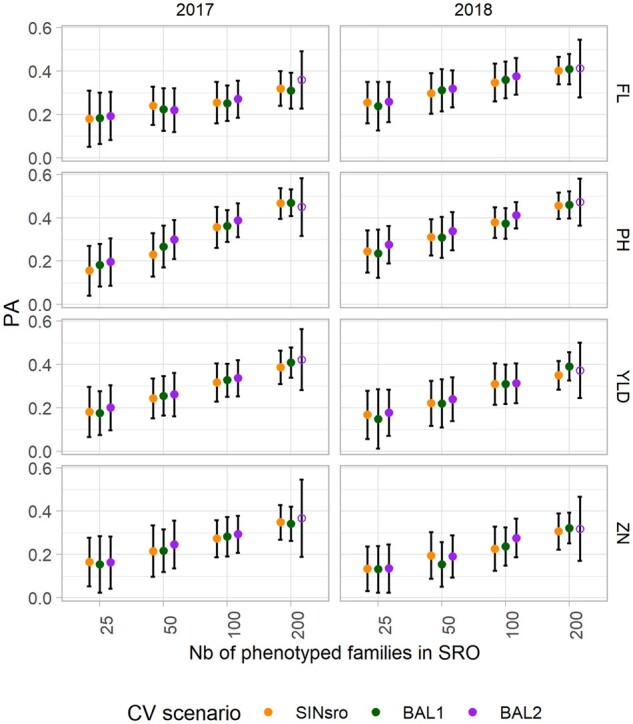
Mean predictive ability (PA) of the GBLUP model to predict phenotypes at Santa Rosa (SRO) for the three CV scenarios: single-site data in SRO (SIN_SRO_) and two-site data with balanced information from the two sites (BAL1 with 100% overlap and BAL2 with 50% overlapped entries). The results for both years (2017 and 2018) and the four traits are presented. The bars represent the standard deviation and the open dots represent the CV obtained from only 34 genotypes.

Two CV scenarios including SRO and PAL (BAL1 and BAL2) were compared with SIN_SRO_ including only SRO data to investigate the combined effect of the training set composition and its size (Figure 5). The calibrations were tested in the two different years for their ability to predict line performance in SRO.

When the two sites were included in the training set, the main source of variation was the number of phenotypes from SRO and PAL included in the training set. Comparing the PA associated with the set size “s” in the case of BAL1 and BAL2 with the PA obtained with the same “s” in SIN_SRO_ allowed us to assess the effect due to the addition of phenotypes from PAL to the training set. Globally, across all set sizes, PA in the BAL2 scenario was greater for all the traits considered (Supplementary data Table S6), with average PA ranging from 0.23 for ZN to 0.38 for PH.

Although training set size was the factor explaining most of PA variation (>22%) for all traits, year effect had some importance (11%) but only for FL. CV methods on the other hand accounted only for a small fraction of the PA variation. The highest gains in PA provided by any two-site CV scenarios compared with the single-site model were obtained for the training set size of 50 to predict PH_2017 (PA increase of +0.07) using the BAL2 model.

### Two-site calibration as a sparse testing approach

So far, we have compared single-site prediction with two-site prediction methods to predict the phenotype of families that were never observed, based solely on between-family information exchange. Another possible approach is to take advantage of the population information by phenotyping all the families in one environment other than the one targeted for the prediction. As PAL is easier to manage, being free of main rice pathogens and closer to the research institute, we tested a scenario with unbalanced representation of the sites in the training sets (IMB), where all 334 families phenotyped in PAL and only a subset of a varying set size “s” phenotyped in SRO were considered.

**Figure 6:**
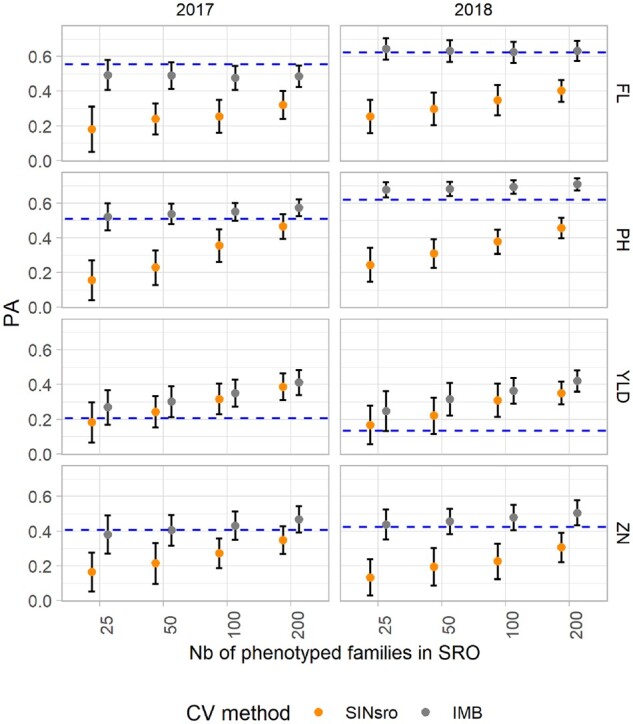
Mean predictive ability (PA) of the GBLUP model to predict phenotypes at Santa Rosa (SRO) for two CV scenarios: single-site data in SRO (SIN_SRO_) and two-site data with complete information in Palmira and incomplete in target site SRO (IMB). The results for both years (2017 and 2018) and the four traits are presented. The bars represent the standard deviation. Dotted blue lines indicate the phenotypic correlation between sites.

The PA were improved by including the phenotypes of the whole population in PAL in the training set, and this was consistently observed for all traits, although to a different extent (Figure 6 and Supplementary Table S7). The largest differences in average PA were observed for FL (SIN_SRO_ = 0.29, IMB = 0.56) and PH (SIN_SRO_ = 0.33, IMB = 0.62). However, for both traits the increase of “s” did not yield a much higher PA with the IMB method. Average ZN predictions also benefited from PAL information, but less so (SIN_SRO_ = 0.24, IMB = 0.45). For those three traits, the average PA with the IMB method was rather close to the phenotypic correlation between the two sites (dotted line in Figure 6). Conversely, for YLD the average PA was similar between SIN_SRO_ (0.27) and IMB (0.34), with values above the indirect phenotypic prediction as represented by the site correlation. The partition of factor effects in the linear model revealed that the proportion of variance explained by the CV method depended on the traits (7% for YLD compared with ≥50% for all other traits; Table 5). Only for YLD did the set size account for a large fraction (32%) of the explained PA variance. The contribution of the year effect to the total PA variance was low (≤1%) for YLD and ZN while still contributing to a small portion of the variance for FL and PH (10% and 6.5%, respectively). For both traits, average PA was higher in the S_0:3_ 2018 trials.

**Table 5:** Analysis by trait of the factors influencing the variability of PA

		SIN_SRO_/BAL1/BAL2		SIN_SRO_/IMB	
Trait^*a*^	Factor^b^	Eta^2^	*R* ^2^	Eta^2^	*R* ^2^
FL	CV	0.003	0.342	0.619	0.792
	Year	0.116		0.104	
	Set size	0.215		0.020	
	CV: year	0.000		0.010	
	CV: set size	0.002		0.026	
	Year: set size	0.003		0.000	
	CV: year: set size	0.003		0.000	
PH	CV	0.009	0.539	0.620	0.853
	Year	0.019		0.065	
	Set size	0.492		0.094	
	CV: year	0.001		0.018	
	CV: set size	0.004		0.050	
	Year: set size	0.012		0.004	
	CV: year: set size	0.002		0.002	
YLD	CV	0.004	0.407	0.072	0.404
	Year	0.009		0.001	
	Set size	0.390		0.319	
	CV: year	0.000		0.003	
	CV: set size	0.003		0.006	
	Year: set size	0.001		0.001	
	CV: year: set size	0.001		0.002	
ZN	CV	0.004	0.322	0.499	0.630
	Year	0.020		0.001	
	Set size	0.291		0.096	
	CV: year	0.000		0.019	
	CV: Set size	0.003		0.015	
	Year: Set size	0.001		0.001	
	CV: Year: Set size	0.003		0.000	

The data are the PA for the CV scenarios comparing SIN_SRO_, BAL1 and BAL2, or SIN_SRO_ and IMB. Eta^2^ is the proportion of variance associated with each effect and *R*^2^ is the coefficient of determination obtained from a linear model applied to the data from the 100 iterations (*n* = 2400 for the model including SIN_SRO_, BAL1 and BAL2 scenarios and *n* = 1600 for the model including SIN_SRO_ and IMB scenarios).

a Traits are days to flowering (FL), plant height (PH), grain yield per plot (YLD), and grain Zn concentration (ZN).

b Factors are CV (SIN_SRO_, BAL1, BAL2, and IMB), Year (2017 and 2018)and set size (25, 50, 100, and 200).

## Discussion

### Evaluation of early generation progenies

The training population with which we tested the various CV scenarios had the expected characteristics for applying GP, both in terms of marker density relative to the specific population LD and total absence of structure among the 334 S_0_ genotypes (Appendix A and Supplementary Tables and Figures).

Our progeny phenotyping method could not capture the within-line variation, as we recorded traits as the mean of the evaluated plot (FL and PH) or from the bulked harvested plot (YLD and ZN). For most combinations of traits and sites, the difference in *H*^2^ between the S_0:2_ and S_0:3_ progeny testing was limited and fell within the confidence interval of each other. However, the *H*^2^ of the S_0:2_ progenies was significantly higher for FL and YLD in SRO and was significantly lower for PH in PAL. This lack of consistency suggested that changes were driven more by environmental causes than by the degree of allelic fixation within the genetic material. This was supported by the temporal checks for which a significant year effect could be observed for all traits and sites, while no effect of the generation was observed. We concluded that the changes in mean between S_0:2_ and S_0:3_ within sites were essentially driven by the environment effect. As the phenotypic variance due to generation was minor compared with the variance associated with the year, generation advance did not seem to influence the PA. For time and economic reasons, calibration on S_0:2_ phenotypes could thus be preferred as it allows a reduction of the breeding cycle length and cost.

### Potential of early GP

We first tested GP models on the early generation phenotypes collected in a single environment. As expected, regardless of the generation, the four traits showed differences in mean PA. FL and PH were overall the best predicted traits, followed by ZN and YLD. This was fairly consistent with what is reported in the literature where FL and PH generally show high PA in absolute terms and relative to yield parameters (Combs and Bernardo 2013; Spindel *et al.* 2015; Ben Hassen *et al.* 2018a,b). However, when comparing with another GP study performed on families derived from rice synthetic populations much higher PA for FL was achieved than in Grenier *et al.* (2015), where average PA for FL reached only a maximum of 0.29 for the population of 343 S_2:4_ lines. Conversely, maximum PA for PH (0.46) was comparable with the PA obtained for the 343 S_2:4_ (0.50) (Grenier *et al.* 2015), but lower than the PA obtained for the 174 S_1:3_ (0.52) (Morais Júnior *et al.* 2018). The maximum PA for YLD (0.39) was slightly higher than the maximum reported for the rice diversity panel of 369 elite breeding lines evaluated in replicated yield trials (0.30) (Spindel *et al.* 2015), but lower than that reported for the 174 S_1:3_ lines (0.44) (Morais Júnior *et al.* 2018), despite an *H*^2^ for YLD that was higher in our study (*H*^2^ = 0.58) than in the two others aforementioned (0.44 in S_1:3_ lines and 0.32 in the diversity panel). Overall, for these commonly reported traits, the PA obtained in our study did not greatly differ from what was reported for GP in rice diversity panels or synthetic populations (as reviewed in Ahmadi *et al.* 2020).

Although various studies on maize and spring wheat have proven the effectiveness of the GP-based approach for kernel zinc concentration, to our knowledge no study applying GP to rice for grain zinc concentration has yet been reported. Grain zinc concentration is a complex trait greatly influenced by soil and other associated factors (Jin *et al.* 2013; Hindu *et al.* 2018; Velu *et al.* 2018; Naik *et al.* 2020), so there are great hopes that GP will simplify the process of breeding rice for nutritional quality. On average, the PA for ZN in a single environment was low (0.26 and 0.24, for 2017 and 2018, respectively). However, the maximum PA in SIN_SRO_ reached 0.36 with 200 S_0:2_ progenies (2017 data and RKHS model), which is comparable to the average estimated PA obtained with the fivefold CV1 model applied to the HarvestPlus association mapping panel of 330 wheat lines (PA = 0.36; Velu *et al.* 2018).

### Effect of the GP methods on PA

In the context of single-site analysis, we found that the two prediction methods, GBLUP and RHKS, induced some differences in PA only for FL. Although GBLUP uses a linear kernel that models only the additive effects, RKHS uses a Gaussian kernel that carries the additive effects and the additive–additive epistatic effects at every possible order (Jiang and Reif 2015). RKHS has been reported to perform better than the linear model in the presence of epistasis (González-Camacho *et al.* 2012; Jiang and Reif 2015; Onogi *et al.* 2015). Epistasis has been reported in FL (Hori *et al.* 2016), PH (Yu *et al.* 2002; Shen *et al.* 2014), YLD (Luo *et al.* 2001; Xing *et al.* 2002), and ZN (Lu *et al.* 2008; Norton *et al.* 2010); however, both GP methods performed similarly for the traits we looked at in our population. The phenotypes we considered were all progeny means, which represent the breeding value or additive effect of our tested S_0_ (Falconer 1960). Different and opposed epistatic effects can appear in the same family and have probably impeded RKHS from capturing them accurately. Limited differences between the two GP methods have also been reported in previous studies testing predictions for rice collections of fixed accessions (reviewed by Ahmadi *et al.* 2020) or S_1:3_ lines extracted from synthetic populations (Morais Júnior *et al.* 2018). Given our phenotypes and considering the PA, GBLUP appeared as the most appropriate method in our context of population breeding considering single or two-site phenotyping data in our calibration models.

### Prediction of the target environment using the two-site calibration model

Most of the contrasts in phenotypic records observed between the two sites were due in large part to the differences in crop establishment, soil conditions, climatic, and biotic constraints as well as field management. Between irrigated and rainfed conditions, not only yield performance was expected to be affected by the environmental conditions, but also the grain zinc concentration, these two traits showing lower correlation between sites. Under flooded conditions, the soil oxygen and redox potential will drop and trigger the formation of non-available zinc or its adsorption onto different compounds, depending on the soil type (reviewed in Rehman *et al.* 2012). As PAL is subject to continuous flooding, low zinc availability was expected and, consequently, observed ZN was much lower than in SRO.

Knowing the environment effect on the trait expressions and the phenotypic correlation between the sites, we tested the potential of GP including two sites with various CV schemes involving several factors. Of all the factors tested in the scenarios, the training set size had the most influence on PA. Training set size explained most of the differences observed for all the traits. The year of phenotyping was best in explaining the PA variations only for FL, which could be related to climatic differences and/or small changes in crop establishment date, which both are known to affect crop phenology. The CV methods SIN_SRO_, BAL1, and BAL 2, accounted for a small portion of the variance explained by the models. In general, the two BAL scenarios showed a limited advantage over SIN_SRO_ for all traits. The prediction of unobserved genotypes for a specific environment using a two-site model was as precise as that obtained with a single-site model. Indeed, the prediction of ${gs}_{\mathrm{SRO}}$ is based on the same amount of information as the $g_{k}$ from a SIN_SRO_ calibration. For this reason, the two-site calibration could perform better only if $g_{k}$ is more precise and relatively larger (larger associated variance) than ${gs}_{\mathrm{SRO}}$, but this is expected only for well-correlated environments. BAL1 and BAL2 differed in the number of genotypes repeated over the two sites. In BAL1, 100% of the genotypes included in the calibration had phenotypes in both sites (the overlapping proportion), whereas only 50% of the included genotypes had phenotypes of both sites in BAL2. The effect of the overlap proportion was tested by Jarquín *et al.* (2020) in a study that assessed the effects of data allocation on the PA of genomic-enabled prediction models. With their GxE model (M3 as presented in their article), the use of overlapping sets of genotypes improved the precision. In our case, the tendency was the reverse. Maintaining similar efforts in phenotyping in both sites while reducing the overlap (BAL1 and BAL2 with the same “s” progenies) resulted in higher precision in the predictions, but only for a specific case of PH_2017 with small training set size of 50 genotypes. For PH, exploring more of the population genetic variability within relatively small training set sizes might have had a greater impact thanks to a higher phenotypic correlation between sites.

Although neither BAL1 nor BAL2 could greatly improve PA compared with SIN_SRO_, calibrating with the whole population phenotyped in PAL and only a subset “s” of the population in SRO for predicting in SRO (IMB) generated substantial improvement of PA for all traits. The interest of this sparse testing method lies in borrowing information within lines across environments (Lopez-Cruz *et al.* 2015). However, if the phenotypes are not correlated between sites, benefits from the inclusion of both environments are expected to be low, as we found with YLD, where less improvement of PA was achieved through IMB than for the other traits. Generally, sparse testing is in most cases more precise than the prediction of unobserved genotypes in known environments, regardless of the calibration method used (Burgueño *et al.* 2012; Jarquín *et al.* 2014; Lopez-Cruz *et al.* 2015; Ben Hassen *et al.* 2018a; Millet *et al.* 2019). However, as the predicted lines must be observed in at least one environment, the burden on the phenotyping still remains, but the effort can lead to an increase in PA for traits with strong to moderate environment correlation, as was the case for FL, PH, and ZN. For ZN, which has only a moderate site correlation, the IMB yielded a large gain in PA even with a drastic reduction of the phenotyping effort in SRO (from SIN_SRO__s25 = 0.14 to IMB_s25 = 0.44 with the 2018 data). Overall, the sparse testing provided an improvement in the prediction of ZN in the rice synthetic population, with average PA (IMB_s200 = 0.51 with the 2018 data) in the range of those reported for spring wheat (Velu *et al*. 2016 ) and maize (Mageto *et al.* 2020).

### Optimization of calibration procedure for GP

In our study, we tested the calibration of a GP model using phenotypic records gathered from early progeny testing in two sites. The potential of using two-site data and sparse testing for the model calibration, was considered as a satisfactory measure to predict most traits, even for YLD, despite a slightly reduced advantage compared with what was reported for the other traits.

We have demonstrated that the calibration using phenotypic data collected on progeny testing at two successive early generations could deliver relatively good and comparable PA. This opens up possibilities for rapid cycling RS, with recycling of parental lines from the genotyping of S_0_ plants, based on the breeding value of the S_0_. Yet, there is still a need to confirm that the models do predict well the performance of more advanced generations for inbred line development. Indeed, the units to derive in the pedigree breeding scheme should be selected on the basis of “varietal ability” (Gallais 1979), which is the expected value of all lines within a family at fixation. This will be explored in our next study, with an external validation of the GP models using a different set of S_0_ progenies extracted from the PCT27 and brought to near fixation.

We are aware that the optimized scheme we suggest, based on random sampling of the training set, genome-wide markers considered as random effects, and random allocation of genotypes to sparse testing could be improved further still by considering other criteria known to increase the performance of GP. It remains to be seen whether PA can be improved by optimized assembly of the training set as performed in various studies (Rincent *et al.* 2012, 2017; Bustos-Korts *et al.* 2016; Akdemir and Isidro-Sánchez 2019; Mangin *et al.* 2019), by inclusion of particular weights for some specific loci (Spindel *et al.* 2016; Bhandari *et al.* 2019; Frouin *et al.* 2019) or by use of an efficient method to proceed to sparse testing in the context of GxE models (Ahmadi *et al.* 2020).

Notwithstanding optimization of the calibration to develop efficient prediction models to fit our scheme, we ought also to consider the gain of applying GP-aided RS in our rice breeding program. So far, only the PA within generations has been tested, starting with the extraction of S_0_ fertile plants of the *C_n_* cycle. Prediction of S_0_ in *C_n_*_+1_ would be done with calibration based on data from the previous cycle *C_n_*. This has been tested through simulation (Müller *et al.* 2017; Ramasubramanian and Beavis 2020) and showed that the persistency of PA across cycles could be achieved with the accumulation of data from several past cycles. Simulation studies will be performed on our population to optimize the long-term use of GP-aided RS and define how and when it is best to upgrade the calibration model. The simulation will also offer the opportunity to improve the prediction and apply genomic selection while maintaining enough genetic diversity for further use of the population.

## Data availability

All supplementary tables, figures, and the data used in this study are available at figshare: https://doi.org/10.25387/g3.14139806.

## Appendix A: “GBS” and data treatment

DNA libraries were prepared at the Regional Genotyping Technology Platform (http://www.gptr-lr-genotypage.com) hosted at Cirad, Montpellier, France. For 949 S_0_ plants extracted from the PCT27, including the 334 considered in the training set, genomic DNA was extracted from the leaf tissues of a single S_0_ plant grown in PAL, using a MATAB lysis buffer (Risterucci *et al.* 2000) and purified using the NucleoMag C-Beads protocol from Macherey-Nagel. Each DNA sample was diluted to 20 ng/μl and 150 ng was digested separately with two restriction enzymes PstI and MseI. DNA libraries were then single-end sequenced in a single-flow cell channel (*i.e.*, 96-plex sequencing) using an Illumina HiSeq2000 (Illumina, Inc.) at the Regional Genotyping Platform (http://get.genotoul.fr/) hosted at INRA, Toulouse, France.

The fastq sequences were aligned to the rice reference genome, Os-Nipponbare-Reference-IRGSP-1.0 (Kawahara *et al.* 2013) using Bowtie2 with the default parameters (option very sensitive). Non-aligning sequences and sequences with multiple positions were discarded. Single-nucleotide polymorphism (SNP) calling was performed using the Tassel GBS pipeline v5.2.29 (Glaubitz *et al.* 2014). The filters applied to loci are the missing data (<20%), the depth for each data point (>10), the minor allele frequency (>2.5%) and the bi-allelic status of SNPs. To limit the probability of under-calling a heterozygous site, the read depth for SNP calling was set to a minimum of 10, so that the probability of under-calling a heterozygous site was limited to a theoretical maximum of 0.2% (Swarts *et al.* 2014). Missing data were imputed using Beagle 4.1 embedded in the R package Synbreed v0.11-22 (Wimmer *et al.* 2012).

After quality control, 9928 SNPs remained for the genetic characterization of the training set and the GP step. All following analyses were thus performed on the 334 S_0_ plants, the latter used in the GP models. Graphical representation of the SNP distribution across the 12 chromosomes was performed using the Synbreed package (Wimmer *et al.* 2012). LD was calculated by computing the pairwise LD measure *r*^2^ as in (Hill and Robertson 1968) with PLINK1.09 using every pair of variants within a 50 variants window (Purcell *et al.* 2007). Non-linear regression modeling was performed using the *nls* function in the statistical package R v3.3.0 (R Core Team 2017) to represent the LD on each chromosome. The effective population size was computed using the linkage disequilibrium method (Hill 1981; Waples 2006) with the software NeEstimator V2.01 (Do *et al.* 2014). Inference of population structure was performed using the *snmf* function from the R package LEA (Frichot and François 2015). Population structure was graphically investigated by first computing Euclidean distances between the genotypes and then building a neighbor joining tree. The computation and graphical representation were done with DARwin V6.0.021 (Perrier and Jacquemoud-Collet 2006).

### Genetic characterization of the population

The 9928 SNP markers were fairly well distributed among the 12 rice chromosomes (Supplementary Figure S1), with an average marker density of one SNP every 40 kb ranging from 27.3 to 64 kb (Supplementary Table S1). For half the markers, the average distance between the nearest neighbors was 9.9 kb, ranging from 3.1 to 15.5 kb according to the chromosomes. The distribution of MAF in the population (Supplementary Table S1 and Figure S2) followed a beta distribution with beta = 5.45 and alpha = 1.37 showing a great proportion of less frequent alleles. Half the loci had an MAF below 15.7%. Across the whole genome, the average heterozygosity per locus was 30%, with loci having a minimum of 2.7 to a maximum of 100% heterozygous genotypes (Supplementary Table S1). The 334 S_0_ genotypes were either relatively fixed (0.08% of heterozygous loci) or fairly heterozygous (41% of heterozygous loci), and half the population was heterozygous for at least 29% of the loci (Supplementary Table S1). The effective population size of the PCT27 measured based on the LD among the 334 S_0_ was *N_e_ =* 40. Pairwise LD in the population across the 12 chromosomes was rather large with an average *r*^2^ of 0.59 for distance between 0 and 25 kb (Supplementary Table S2). The LD decreased to 50% of its initial value at a slow rate (300–400 kb; Supplementary Figure S3). No structure was found in the population, as illustrated by the neighbor joining grouping based on similarity distances (Supplementary Figure S4).

## Appendix B: Analysis of the year and generation effect

For the 334 S_0_ families of the PCT27 used in this work, the generation S_0:2_ was phenotyped in 2017 and the generation S_0:3_ in 2018. To measure the year effect disconnected from the generation effect, 50 families (temporal checks) from the same PCT27 were observed in 2017 and 2018 as generation S_0:2_. Similarly, to evaluate the extent of the generation effect that would result from inbreeding, the same 50 temporal checks were observed as S_0:2_ and S_0:3_ in 2018. This was done for all traits. An alpha-lattice design with eight unbalanced blocks and three replicates was used for each trial.

To reduce the block effect resulting from the sampling of temporal checks, each block was enhanced with two spatial checks (SC), one plot of IR64 (indica mega variety) and one plot of L23 (tropical japonica inbred line from the CIAT-Cirad upland breeding program) and so centered the block value on the SC mean value.

To assess the year effect, the following mixed model was applied by site to the data of the 2 years for the 50 temporal checks at generation S_0:2_ and the two spatial checks.

$Y_{\left\{\mathrm{ijklm}\right\}}=\mu +y_{\left\{i\right\}}+r{\left(y\right)}_{\left\{ij\right\}}+SC{\left(y\right)}_{\left\{ik\right\}}+b{\left(r\left(y\right)\right)}_{\left\{\mathrm{ijl}\right\}}+g_{\left\{m\right\}}+{yg}_{\left\{im\right\}}+\varepsilon_{\left\{i\mathrm{jklm}\right\}}$ , the fixed part of the model was composed of the intercept μ, the year effect y, the replicate effect r and the SC variable, which discriminates the two spatial checks from each other and from the PCT27 lines k={PCT27, IR64, L23}. The random part was composed of the line (genotype) effect g with distribution $g∼\left(0,\;I\sigma_{g}^{2}\right)$, the line by year interaction gy with distribution $yg∼\left(0,\;I\sigma_{yg}^{2}\right)$ and the error ε with distribution $\varepsilon ∼\left(0,\;I\sigma_{\varepsilon }^{2}\right)$.To assess the generation effect, the following model was applied to the data of the 50 temporal checks at generation S_0:2_ and S_0:3_ and the two spatial checks in 2018.

$$Y_{\left\{\mathrm{ijklm}\right\}}=\mu +r_{\left\{i\right\}}+b{\left(r\right)}_{\left\{ij\right\}}+SC{\left(G\right)}_{\left\{kl\right\}}+g_{\left\{m\right\}}+Gg_{\left\{jm\right\}}+\varepsilon_{\left\{\mathrm{ijklm}\right\}}$$

The parameter annotation was the same as for the analysis of the year effect, with additionally G as the fixed effect of the generation and Gg as the random interaction between the line and the generation with distribution $Gg∼\left(0,\;I\sigma_{Gg}^{2}\right)$.

The results can be seen in Supplementary Table S4.

## Appendix C: Phenotypic data preparation

In PAL, where the number of plants was known, any single plot with <14 established plants was identified and the data were removed if it strongly differed from the other two replicates. After this first round of cleaning, a mixed model was applied to each trial separately with the intention to discard the plot phenotypic scores of progenies with inconsistent records among the repetitions within a site, either as a result of poor crop establishment or unexpected problem on the specific plot. Within each trial, the plots with a residual in absolute value at more than four standard residuals were labeled as outliers and removed. The model used to remove outliers was formulated as follows and was the same for each trial:

$$Y_{\mathrm{ijk}}=\mu +r_{i}+b{\left(r\right)}_{ij}+g_{k}+\varepsilon_{\mathrm{ijk}}$$

where μ is the general intercept, r is the fixed effect for the replicates, b is a random block effect nested in r with distribution $b∼N\left(0,I\sigma_{b}^{2}\right)$, g is the random genotype effect with distribution $g∼N\left(0,I\sigma_{g}^{2}\right)$, and ε is the random error term with distribution $\varepsilon ∼N\left(0,I\sigma_{\varepsilon }^{2}\right)$. This model, as well as all the following mixed models was fitted using ASReml-R v3.0 (Butler *et al.* 2007).
